# Winnowing DNA for Rare Sequences: Highly Specific Sequence and Methylation Based Enrichment

**DOI:** 10.1371/journal.pone.0031597

**Published:** 2012-02-15

**Authors:** Jason D. Thompson, Gosuke Shibahara, Sweta Rajan, Joel Pel, Andre Marziali

**Affiliations:** 1 Department of Physics and Astronomy, University of British Columbia, Vancouver, British Columbia, Canada; 2 Boreal Genomics Inc., Vancouver, British Columbia, Canada; Chinese Academy of Science, China

## Abstract

Rare mutations in cell populations are known to be hallmarks of many diseases and cancers. Similarly, differential DNA methylation patterns arise in rare cell populations with diagnostic potential such as fetal cells circulating in maternal blood. Unfortunately, the frequency of alleles with diagnostic potential, relative to wild-type background sequence, is often well below the frequency of errors in currently available methods for sequence analysis, including very high throughput DNA sequencing. We demonstrate a DNA preparation and purification method that through non-linear electrophoretic separation in media containing oligonucleotide probes, achieves 10,000 fold enrichment of target DNA with single nucleotide specificity, and 100 fold enrichment of unmodified methylated DNA differing from the background by the methylation of a single cytosine residue.

## Introduction

Many challenges in clinical diagnostics are rooted in the difficulties of detecting rare molecules. The progress of tumor development can be indicated by rare DNA mutations present in a handful of cells within a larger, wild-type, cell population [1]. Fetal DNA in maternal blood, while in low abundance, can be used to provide an early, non-invasive diagnostic of fetal genetic disease [2]. The presence of even a few cells of a pathogenic organism in human clinical samples can be used to diagnose infections and determine the correct treatment course [3]. Unfortunately, our ability to analyze biological samples for these rare sequence or methylation variants, and subsequently to improve our understanding of human disease progression, enable early diagnosis, and develop more effective treatments, is limited by our capacity to analyze rare variants in a sea of background DNA. While new methods for DNA sequencing promise to allow routine sequencing of clinical samples, the error rates in these methods are much greater than the frequency of many diagnostic sequence variations [1], [4]. Increasing sequencing coverage to target low abundance variants is complicated by the fact that errors in high throughput sequencing methods tend to have substantial sequence bias [4]. As a result, sequencing methods are generally not capable of detecting rare variants whose abundance is more than 100-fold lower than the background sequence [5]. Several methods for the detection of rare sequence variants have been developed based on the polymerase chain reaction (PCR) by employing some means of reducing the amplification efficiency of background sequences [6], [7], or using digital PCR schemes to enumerate the rare variants [8]. These PCR based methods are collectively capable of detecting rare sequence variants whose abundance is between 1,000- and 10,000-fold lower than the most abundant background sequence. This sensitivity is limited by the fidelity of polymerases [9], and by sample volume and total template DNA limitations [10], [11]. The problem of detecting rare variants becomes even more difficult when the target is identical in sequence to the background DNA, differing only in its methylation pattern. In this case detection requires either chemical modification or sequence-independent enrichment of all methylated DNA prior to detection and analysis.

The limitations of existing analytical methods for the detection of rare sequence variants can be overcome by enriching a sample for sequences of interest prior to analysis. Enriching for a low abundance sequence so that it is present at a molar concentration comparable to the most abundant sequence in the sample would enable the detection of even the rarest sequence variants in the sample using any standard analysis method, or would allow analysis of multiple regions through massively parallel sequencing. Existing methods of sequence enrichment rely on sequence-specific hybridization between the target DNA and a complementary synthetic probe DNA [12], [13]. The thermodynamics of DNA hybridization limits these enrichment methods, which rely on a single hybridization event per target molecule, to enrichment factors of 10- to 20-fold [14], [15]. Repeated hybridization-wash cycles can achieve arbitrarily high purification factors, limited only by the number of times one wishes to repeat the cycle [16]. In practice, however, this is time consuming, labor intensive, and leads to losses that scale geometrically with the number of cycles. Such losses are problematic when searching for rare sequence variants since samples will contain, by definition, a limited number of target molecules. Here we present a fundamentally new sample preparation method that combines chemical and physical methods to selectively enrich for low abundance DNA that differs from the background by either a single nucleotide or by the methylation status of a single cytosine residue.

We present a method for hybridization based sequence enrichment which ensures that each target DNA molecule repeatedly interacts with probe oligonucleotides, resulting in highly specific enrichment without the need for user intervention or laborious wash steps. The technique is a new version of Synchronous Coefficient Of Drag Alteration (SCODA) purification. SCODA purifications work by applying a periodic forcing field to drive target molecules in a circular orbit, while synchronously altering the mobility (or equivalently the drag) during part of the forcing cycle, such that during each cycle target molecules experience a net displacement towards the center of a two dimensional domain. After many cycles, target molecules accumulate at a central focus location.

### Background and Theory

The first demonstration of SCODA purification relied on the mobility of DNA migrating through a sieving matrix, such as agarose or polyacrylamide, to be dependent on the magnitude of an applied electric field [17]. By applying an appropriate periodic electric field pattern to a sieving matrix, a convergent velocity field can be generated for all molecules in the gel which have an electric field dependent mobility. For DNA and RNA, this field dependent mobility is a result of the physical interactions between the long charged polymers and the sieving matrix [18]. Most other bio-molecules are either shorter, less charged, or less able to undergo conformational changes, and have field independent mobilities. This allows one to use SCODA to purify DNA from samples as challenging as tar [19]. However, the nature of the physical interaction between DNA and the sieving matrix is independent of sequence. To gain chemical control over enrichment of DNA, that is to be able to select specific DNA sequences to extract, it is necessary to chemically tune the physical response of the DNA as it migrates through the gel. This is achieved through repeated, transient hybridizations of target DNA to complementary oligonucleotide probes immobilized in the gel. The physical passage of target sequences through such a gel will depend both on temperature and the homology between the target and probe sequences. Adjusting the temperature provides a means to alter the mobility that can be exploited with SCODA to enrich for specific targets. Unlike traditional hybridization-based enrichment methods which rely on a single hybridization event per molecule, this enrichment technique ensures that each target molecule undergoes multiple hybridizations to the immobilized probes before reaching the centre of the gel, where each period of the sequence specific SCODA (ssSCODA) cycle is effectively a hybridization-wash cycle. These repeated hybridizations impart this technique with a specificity that, to our knowledge, exceeds all other hybridization based enrichment techniques, and is capable of resolving targets identical in sequence and differing only by the methylation status of a single cytosine residue.

To see how immobilized probes affect the mobility of complementary target DNA we start by describing the target-probe interactions with first order reaction kinetics:
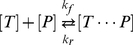
(1)Here 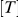 is the target concentration, 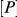 the immobilized probe concentration, 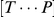 the probe-target duplex, 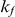 is the forward reaction (hybridization) rate, and 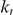 the reverse reaction (dissociation) rate. Since the mobility of the target is zero while it is bound to the matrix, the average mobility of target molecules will be reduced by the relative amount of target that is immobilized on the matrix:
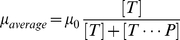
(2)Where 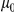 is the mobility of the unbound target. It is possible to approximate the temperature dependence of the average mobility by neglecting the dependence of the forward reaction rate on temperature, and modeling the reverse reaction rate as an Arrhenius barrier crossing. With these two assumptions the mobility can be expressed as: 
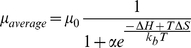
(3)The details of this derivation are presented in File S1. Where 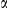 is an empirically derived, dimensionless constant that depends on the details of the forward and reverse reactions. 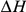 is the enthalpy, 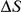 the entropy, 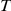 the temperature, and 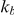 is Boltzmann's constant. This expression describes a sigmoidal temperature dependence where the mobility is the unbound mobility, 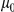, at high temperature, and is zero at low temperature. Equation (2) suggests that the sigmoid is centered on the probe-target melting temperature, which is defined as the temperature where half of the targets are hybridized to probes. Near the melting temperature, a small change in temperature results in a large change in mobility of the target. When an electric field is applied to this type of gel the resulting Joule heating leads to a temperature rise proportional to the square of the magnitude of the applied field. Combining this Joule heating with the temperature dependent mobility described in equation (3) results in a mobility which depends on electric field, enabling one to use SCODA to concentrate and purify complementary targets.

**Figure 1:**
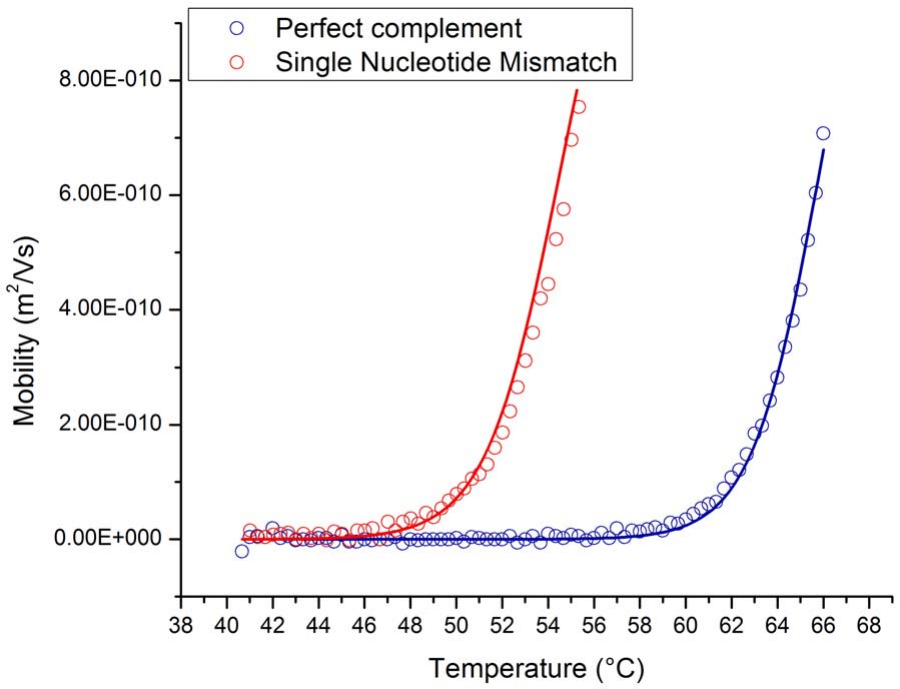
Measurement of temperature dependence of DNA target mobility through a gel containing immobilized complementary oligonucleotide probes. The upper limit of these curves were cut off because target DNA had migrated off the end of the gel used for taking these measurements. Both sets of data points were fit to equation (2) using the non-linear least squares fitting tool in the Origin 7.5 software package (OriginLab Corporation, Northampton MA). Mfold calculated values were used for the enthalpy and entropy terms; μ_0_ and α were determined by the fit. For the mismatch curve μ_o_ = 1.34e-9+/−0.05e-9 m^2^/Vs and α = 1.12e-6+/−0.07e-6. For the perfect complement μ_0_ = 1.28e-9+/−0.05e-9 m^2^/Vs and α = 2.01e-7+/−0.13e-7.

Measurements of mobility as a function of temperature were performed for two fluorescently labeled 100 nucleotide (nt) long oligonucleotides. These targets differed by a single base such that they were either a perfect complement (PC) to a 19 nt gel-immobilized probe or contained a single nucleotide mismatch (snMM). The difference in melting temperature between the two targets and probe was calculated by Mfold [20] to be 9.4°C. Targets were injected into a polyacrylamide gel containing immobilized probes and the gel was exposed to a constant electric field of 25 V/cm while the temperature was ramped with an external heater from 40°C to 70°C at a rate of 0.5°C/min. Images of the gel were taken every 20 sec and subsequently processed to determine the mobility of the oligonucleotides as a function of temperature. The resulting mobility versus temperature curve is shown in Figure 1.

Using values for the binding energy calculated using Mfold, the mobility versus temperature curves were fit to equation (3) and showed good agreement with our model of migration through this type of gel.

## Results and Discussion

### Sequence Specific Concentration and Enrichment

With the temperature dependent mobility shown in Figure 1, sequence specific enrichment of target DNA can be achieved by superimposing a small direct current (DC) bias over the time-varying electric field required for SCODA concentration [17], [19]. The total velocity of a target DNA molecule in the gel is the sum of the SCODA velocity, resulting from the time varying electric field that drives molecules toward the center of the gel, and the velocity due to the applied DC bias. The final focus location of a target molecule will therefore be shifted away from the centre of the gel by an amount which depends on the balance between the SCODA velocity and the DC bias-induced velocity. This displacement depends on the strength of the interaction between the target and probe molecules. As shown in Figure 1, weaker interactions will shift the mobility-temperature curve to the left, resulting in lower SCODA velocity (due to decreased temperature dependence of mobility) and higher DC mobility for a given operating temperature. As a result, weakly interacting mismatched targets are pushed further away from the center of the gel by the applied bias field than more strongly interacting perfect match targets, separating targets into resolvable foci.

**Figure 2:**
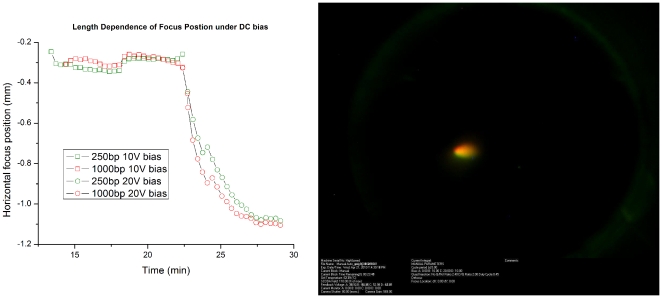
Time series of ssSCODA focusing under bias. PC DNA is tagged with 6-FAM (green) and snMM DNA is tagged with Cy5 (red). Images were taken at 2 min intervals with the first image taken immediately following injection. The camera gain was reduced from 32 to 16 on the green channel after the first image was taken. DNA was injected from a chamber adjacent to the right side of the gel. After injection, focusing plus bias fields are applied. The PC target (green) experiences a convergent drift velocity and focuses to the centre of the gel. The more weakly focusing snMM target (red) is washed completely from the gel by the bias field.

The separation between the PC and snMM velocity versus temperature curves suggest that it should be possible to separate the two species by applying a focusing field combined with a bias field at a temperature of around 62°C, where the mobility of the snMM target is high, but the PC target is only just beginning to become mobile in the gel. To demonstrate separation with single nucleotide specificity, 1 pmol of each target,were mixed into a 250 µl volume and subsequently electrokinetically injected into a gel. Once injected, a superposition of time varying SCODA, and DC bias fields were applied at 62°C. Figure 2 shows images of the purification process taken every 2 min. The PC target was tagged with 6-FAM and shown in green, and the snMM target was tagged with Cy5 and is shown in red (experiments were performed with the dyes reversed with identical results).

It is clear from the images shown in Figure 2 that there are two distinct velocity profiles generated for the two different sequences of target DNA. The PC target converges towards a zero velocity point near the center of the gel, while the snMM target has no zero velocity point within the gel and is washed completely from the gel.

**Figure 3:**
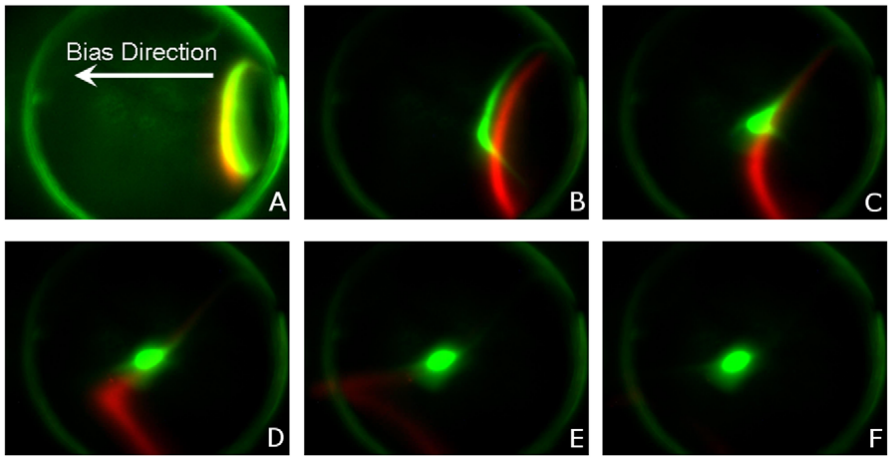
Demonstration of length independent focusing. Left: Focus location under bias for 250 bp (green) and 1000 bp (red) fragments. Right: Image of the gel at the end of the run. Green and red channels have been superimposed.

To measure the length dependence of the final focus location, two different lengths of target DNA, each containing a sequence complementary to gel immobilized probes, were focused under bias and the final focus location measured and compared. The target DNA was created by PCR amplification of a region of pUC19 that contains a sequence complementary to the probe sequence used to generate Figure 1 and Figure 2. Two reactions were performed with a common forward primer, and reverse primers were chosen to generate a 250 bp amplicon and a 1000 bp amplicon. The forward primers were fluorescently labeled with 6-FAM and Cy5 for the 250 bp and 1000 bp fragments respectively. The targets were injected into an ssSCODA gel and focused to the centre before applying a bias field. A bias field of 10 V/cm was superimposed over 120 V/cm focusing fields for 10 min at which point the bias was increased to 20 V/cm for an additional 7 min. Images of the gel were taken every 20 sec, with a 1 sec delay between the 6-FAM channel and the Cy5 channel. The field rotation period was 5 sec. Images were post processed to determine the focus location of each fragment. Figure 3 shows the focus location versus time for the 250 bp (green) and 1000 bp (red) fragments. The left panel is an image of final focus of the two fragments at the end of the experiment. There is a small difference in final location that can be attributed to the fact that the two images were not taken at the same phase in the SCODA cycle. This result shows that the final focus position does not depend on length and has important practical implications, as it suggests that ssSCODA is capable of distinguishing nucleic acid targets by sequence alone without the need for ensuring that all targets are of a similar length.

### Single Nucleotide Enrichment Specificity

**Figure 4:**
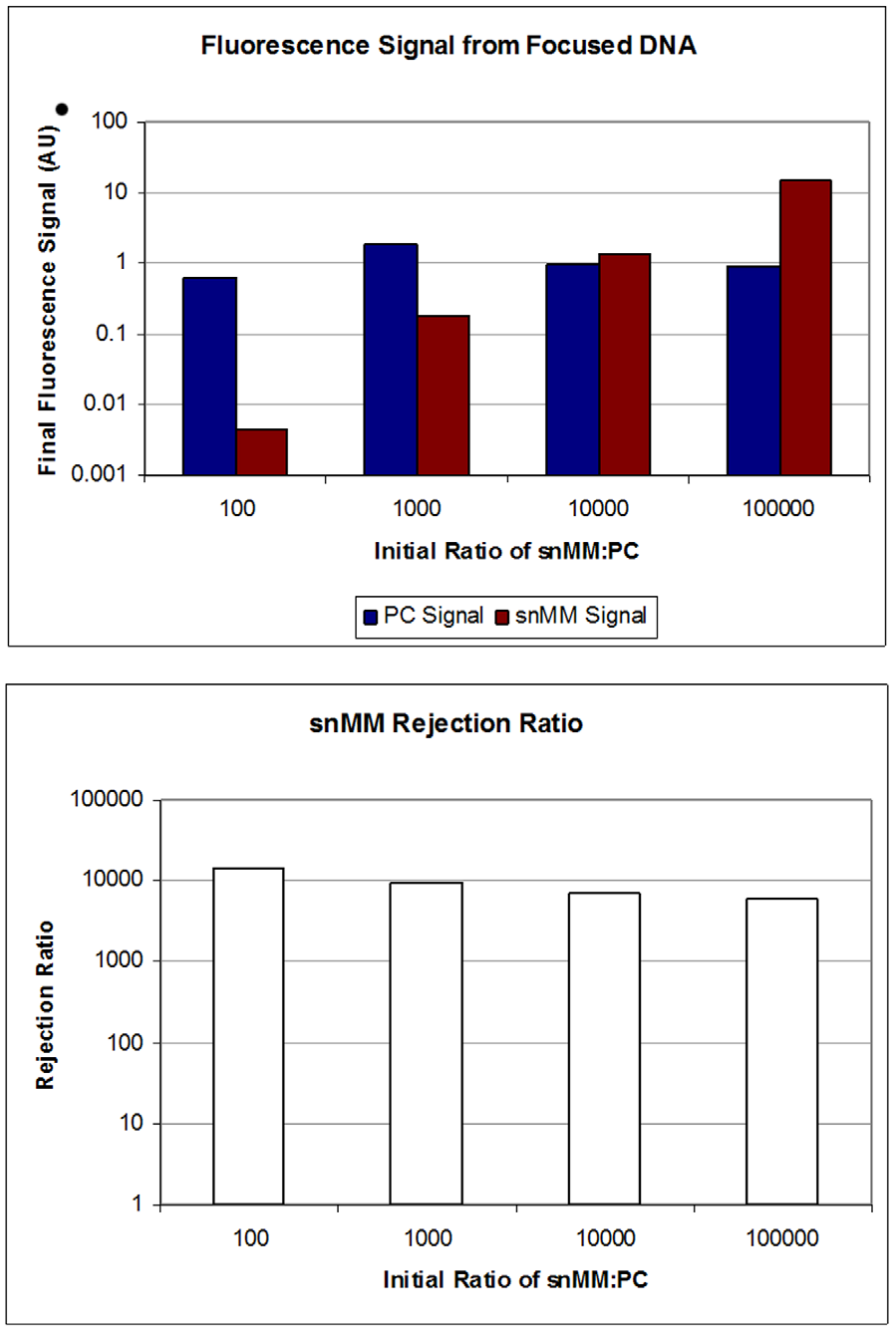
Rejection ratio of snMM DNA. Four different ratios of snMM:PC were injected into a gel and focused under bias to remove excess snMM. The PC DNA was tagged with 6-FAM and the snMM DNA was tagged with Cy5. Top: fluorescence signal from the final focus spot after excess snMM DNA had been washed from the gel. The fluorescence signals are normalized to the fluorescence measured on an initial calibration run where a 1∶1 ratio of PC-FAM:PC-Cy5 DNA was injected and focused to the centre of the gel. Bottom: rejection ratios calculated by dividing the initial ratio of snMM:PC by the final ratio after washing.

To determine the specificity of ssSCODA with respect to rejection of sequences differing by a single base, different molar ratios of the synthetic PC and snMM 100 nt oligonucleotides used to generate Figure 1 and Figure 2, were injected into a gel containing immobilized probes and subsequently focused with an applied DC bias field to remove the excess snMM DNA. After washing the snMM target from the gel, the amount of fluorescence at the focus location was quantified for each dye. By dividing the ratio of snMM:PC injected by the ratio of snMM:PC remaining after wash, the rejection ratio can be determined. The details of how these measurements were made are discussed in File S1. Rejection ratios measured in this manner are shown in Figure 4.

It was found that rejection ratios of at least 10,000 fold are achievable. These final rejection ratios, however, do not tell the full story of the enrichment. Analysis of images during focusing and washing at high snMM:PC ratios suggest that there were snMM molecules with two distinct velocity profiles. Most of the mismatch target washed cleanly off of the gel while a small amount was captured at the focus. These final focus spots visible on the Cy5 channel likely consisted of Cy5 labeled targets which were incorrectly synthesized with the PC sequence due to single base substitution errors. The 10,000∶1 rejection ratio measured here is consistent with estimates of oligonucleotide synthesis error rates with respect to single base substitutions [21], meaning that the synthetic mismatch sample likely contains approximately 1 part in 10,000 perfect complement molecules. This implies that the residual fluorescence detected on the Cy5 channel, which we attribute to unresolved mismatch may in fact be Cy5-labeled perfect complement that has been enriched from the mismatch sample. Consequently the rejection ratio of ssSCODA may actually be substantially higher than 10,000∶1. It should also be noted that this rejection ratio does not depend significantly on the synthesis fidelity of the oligonucleotide probes immobilized in the gel. Incorrectly synthesized probes will be immobilized in the gel at such a low concentration that they will not significantly perturb the velocity of the target DNA.

### Mutation Enrichment

**Figure 5:**
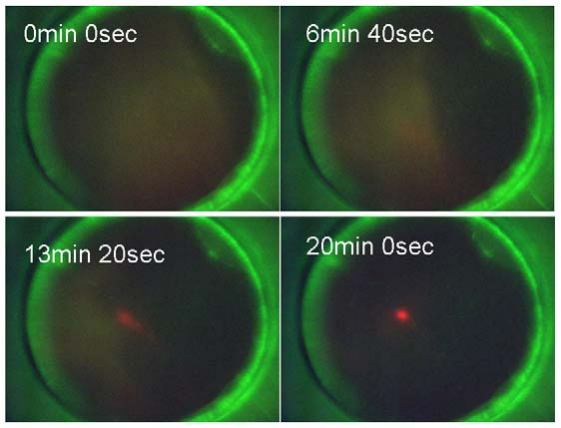
Enrichment of EZH2 Y641N mutation from a 1∶1 mixture of wild type (green) and mutant (red) amplicons. 30 ng of each 470 bp target was diluted into a 250 µl solution of 0.9 mM tris, 0.9 mM boric acid and 2 mM NaCl. The sample was placed in a boiling water bath for 5 min to denature the double stranded DNA prior to injection. The targets were injected from a chamber adjacent to the lower right corner of the gel. After injection, focusing and bias fields were applied to simultaneously concentrate the mutant amplicon while washing the wild type amplicon from the gel.

The ability of ssSCODA to enrich for sequences of significance to disease diagnosis has also been explored. In these experiments cDNA was isolated from cell lines which contained either a wild type version of the EZH2 gene or a Y641N mutant which has previously been shown to be implicated in B-cell non-Hodgkin Lymphoma [22]. 460 bp regions of the EZH2 cDNA which contained the mutation site were PCR-amplified using fluorescent primers in order to visualize concentration and washing. 30 ng of each amplified allele was mixed together and denatured by boiling prior to injecting into an ssSCODA gel and separating. This is shown in Figure 5.

The difference in melting temperature between the wild type (T_m_ = 53.0°C) and mutant (T_m_ = 59.3°C) alleles, as calculated by the Mfold two state hybridization server is 6.3°C, approximately 33% lower than the difference for the oligonucleotides separated in Figure 2. Despite the smaller difference in melting temperatures, the behavior of these amplicons is qualitatively similar to the higher melting temperature difference oligomers shown in Figure 2. The wild type (mismatch) target is completely washed from the gel while the mutant (perfect complement) is driven towards the center of the gel.

The maximum rejection ratio one can measure with this system is limited by the ability to detect the complementary sequence. With our imaging hardware the limit of detection was approximately 10 ng of singly labeled 500 bp amplicons. To repeat the 10,000 fold enrichment shown earlier one would need to mix 10 ng of the mutant target with 100 µg of wild type, which was not practical to generate with PCR. For this reason it was not possible to repeat the 10,000 fold rejection experiments with these targets.

### Methylation Enrichment

In addition to enriching for mutant sequences with single nucleotide resolution we have also demonstrated that this version of SCODA can enrich for low abundance targets based on methylation status. This is accomplished by exploiting the small binding energy difference between a methylated versus unmethylated target when hybridized to a complementary probe. It has been shown that methylation of cytosine residues increases the binding energy of hybridization relative to unmethylated duplexes [23]–[25] resulting in a small increase in duplex melting temperature of about 0.7°C per methylation site. To explore the possibility of exploiting this small difference in melting temperature to enrich for methylated targets, fluorescently tagged PC oligonucleotides were synthesized with a single methylated cytosine residue within the capture probe region and mobility versus temperature curves were generated for methylated and unmethylated versions of the PC target.

**Figure 6:**
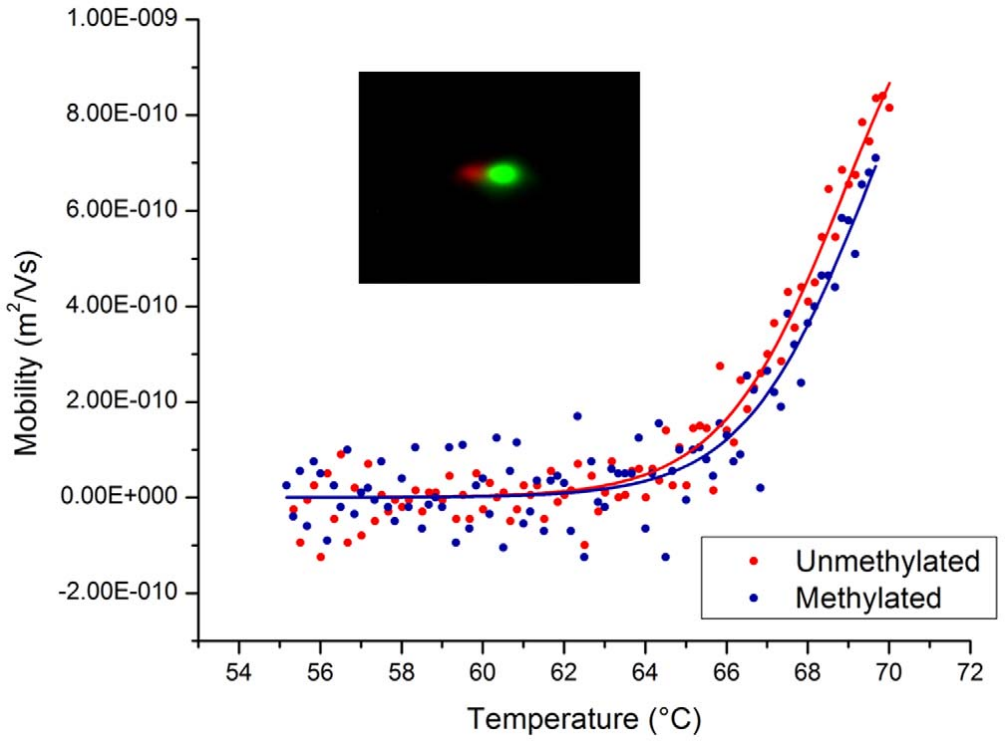
Measurement of mobility versus temperature for methylated and unmethylated targets. Data points were fit to equation (3). The upper limit of these curves were cut off because target DNA had migrated off the end of the gel used for taking these measurements. Both sets of data points were fit to equation (2) using the non-linear least squares fitting tool in the Origin 7.5 software package (OriginLab Corporation, Northampton MA). For the unmethylated curve Mfold calculated values were used for the enthalpy and entropy terms; μ_0_ and α were determined by the fit. Using ΔH = 144.4 kcal/mol and ΔS = 0.3988 kcal/mol K the unmethylated curve fit resulted in values of μ_o_ = 1.34e-9+/−0.05e-9 m^2^/Vs and α = 1.12e-6+/−0.07e-6. For the methylated curve, it was assumed that the parameters μ_0_, α and ΔS were unaffected by the addition of the methyl group and the parameters obtained in the unmethylated fit were used to obtain a value for ΔH = 144.62+/−0.04 kcal/mol. Inset: Separation of methylated (6-FAM, green) and unmethylated (Cy5, red) targets by focusing with an applied DC bias at 69°C.

The mobility versus temperature measurements shown in Figure 6 confirm that there is a subtle difference in binding energy due to the methylation of a single cytosine residue. Figure 6 can also be used to determine the optimal temperature for separating these two targets. Since under bias, the final focus location will be a balance between the inward SCODA velocity and the applied DC bias velocity, the final focus position of the two fragments should have the greatest difference at the temperature where their mobilities have the greatest difference. This temperature can be found by finding the maximal difference between the two expressions used to fit the data points in Figure 6. This was found to be 69°C. The inset of Figure 6 shows separation of methylated and unmethylated targets while focusing using SCODA with a superimposed DC bias at 69°C.

**Figure 7:**
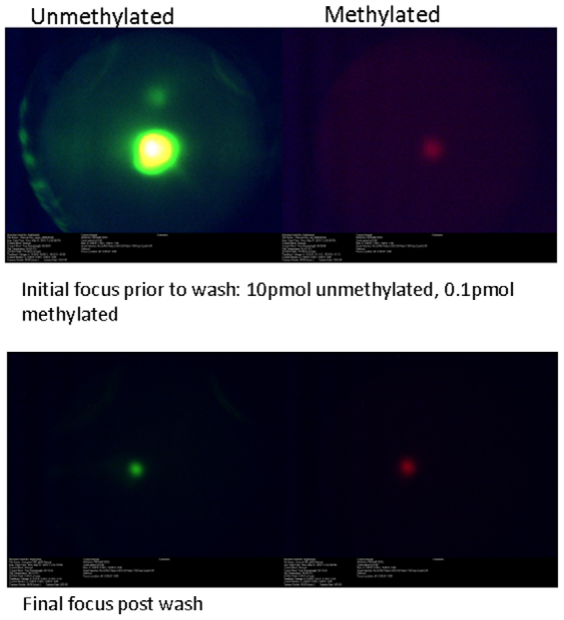
Washing of unmethylated DNA from the gel. Top two images were taken after an initial focus but before attempts to wash. The bottom two images were taken after washing the unmethylated target from the gel. All images were taken with the same gain and shutter settings which resulted in sensor saturation and some ghost images from reflections off of lenses in the top left image. Note that the dyes have been swapped compared to the image in Figure 7.

To determine the ability of ssSCODA to enrich for methylated DNA and extend the applicability of SCODA to epigenetics, a mixture of 1 pmol of methylated target and 100 pmol of the unmethylated target was diluted into 250 µl, electrokinetically injected into the gel and focused to the center without any bias fields applied. The targets were then focused with a bias field to remove the unmethylated target, and finally focused to the center of the gel again, without bias fields, for fluorescence quantification. Fluorescence quantification of these images, shown in Figure 7, indicate that the enrichment factor was 102-fold with losses of the methylated target during washing of 20%.

### Sample Extraction

**Figure 8:**
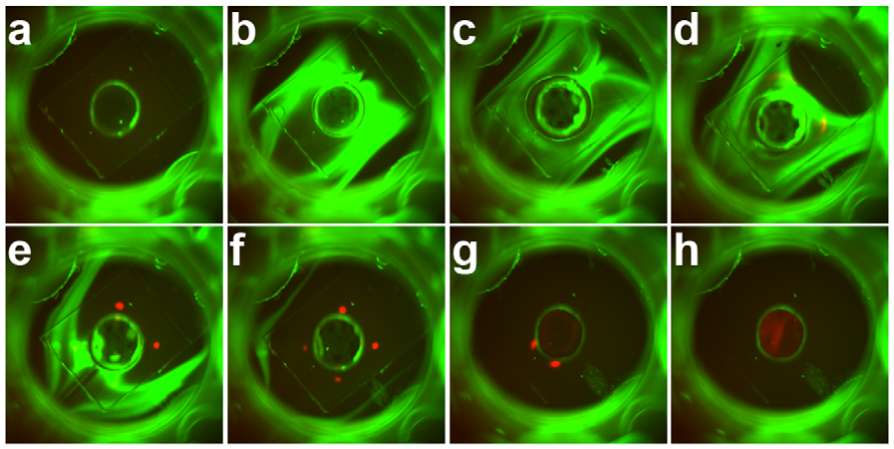
Time series of a demonstration of enrichment and extraction of target DNA fragments from a mixture of 20 fmol of 100 nt target (shown in red) and 1 µg of non-target 460 bp dsDNA (shown in green). The original images in the green and red channels are in gray scale; a–c. The sample mixture is electrophoretically injected from the sample chamber on the bottom left towards to top right. d–g. A focusing field and a time-multiplexed DC bias field is applied to focus the target fragments (red) while washing the background (green) back towards the bottom left. The extraction well in the center is still empty, though condensation on the walls of the well can be observed. h. The extraction well is filled with 12 µl of buffer and only the focusing field is applied. The target fragments enter the buffer in the extraction well. Once focusing is complete, the output buffer can be collected with a pipettor.

In order to extract purified DNA from a ssSCODA gel for subsequent analysis a gel is cast with a hole in the centre in a manner similar to previous implementations of SCODA [19], [26]. Prior to injection of DNA into the gel the extraction well is initially empty (no buffer or gel) and covered with PCR sealing tape to prevent the gel drying out. Once the DNA sample has been injected the rotating SCODA fields are applied with a superimposed DC bias, simultaneously concentrating the target DNA while washing mismatched DNA from the gel. Once the mismatched DNA has been washed from the gel, liquid is added to the central extraction well and the rotating fields are applied for a short time without bias, driving the target DNA into the extraction well where it can be removed. This process is illustrated in Figure 8, where a target containing a sequence perfectly complementary to the gel bound oligos is concentrated while a non-complementary sequence is washed from the gel. The final output volume is around 6 to 12 µl depending on the protocol.

### Rejection of Genomic DNA

With this extraction technique we were able to measure the ability of ssSCODA to reject genomic DNA. An *E. coli* culture was spiked into Human whole blood such that there were estimated to be approximately 10 times more copies of human genomic DNA than *E. coli*, and the mixture was lysed and purified for DNA, sheared to below 1.5 kbp and diluted to 5 ng/µl. Shearing was done to ensure that the DNA fragments could migrate into the polyacrylamide gel. 1 µg (200 µl) of the sample was loaded into an ssSCODA cassette whose focusing gel contained 20 nt-long capture probes complementary to a section in the UidA gene of *E. coli*. The cassette was placed in an ssSCODA instrument and a protocol similar to the one described in Figure 8 was applied to focus the UidA gene while rejecting all other fragments. Once complete, the ssSCODA input and output were quantified for the UidA gene in a qPCR assay to determine target recovery, and for the GAPDH gene in a separate qPCR assay to estimate the background carry through. See Methods and File S1 for details on sample preparation, ssSCODA conditions and protocol, and qPCR assay. We found that the input had a 23-fold excess of GAPDH gene over UidA gene by copy number, and the yields for UidA gene and GAPDH gene were estimated to be 20% and less than 0.12%, respectively, for an enrichment factor of at least 152-fold by copy number. These values are our best estimates because the UidA copy number in the input and the GAPDH copy number in the output were below the limit-of-quantification of the qPCR assay. Extrapolating the standard curves suggests an enrichment factor on the order of 10^3^, however this estimate has considerable uncertainty.

### Conclusions

By chemically tuning the interactions between target DNA and a gel containing immobilized oligonucleotide probes, we have been able to demonstrate a fundamentally new version of DNA purification that can exquisitely select for sequence content. Due to the nature of the underlying SCODA method, each target interacts with immobilized probes multiple times before reaching the central focus location in the gel, a feature which imparts this purification method with outstanding specificity.

This work has demonstrated the ability to perform at least 10,000-fold enrichment of a target sequence in the presence of excess contaminating sequences differing by a single base. Additionally, the target sequence is concentrated 25,000-fold from an input volume of 250 µL to an output volume of 10 nL (based on the diameter of the final focus spot and the thickness of the gel). It should be noted that there is no fundamental limit to the input volume, as larger input volumes simply require more time to electrokinetically inject target DNA into the ssSCODA gel. Performing this level of enrichment has the effect of reducing the sequence complexity of the template DNA, which relaxes the specificity requirements of the ultimate detection method. The level of concentration achievable with this technique promises to improve sensitivity of detection by enabling one to sample larger input volumes.

In addition to the exceptional sequence enrichment performance, ssSCODA has proven to have the unique capability of enriching for target sequences based on methylation state. The repeated target-probe interactions can leverage the small increase in binding energy imparted to a target-probe duplex by a single methylated cytosine residue to enrich for a methylated target. It was demonstrated that ssSCODA is capable of 100-fold enrichment of a methylated target out of a background containing a 100-fold excess of unmethylated targets which are identical in sequence. To our knowledge this is the first demonstration of hybridization-based enrichment of unmodified methylated DNA.

Finally, we have shown that the presence of both E. coli and human genomic DNA does not significantly perturb the ability of ssSCODA to enrich for a specific sequence of DNA. Taken together, we feel that these demonstrations suggest that ssSCODA has the potential to become a very powerful tool for highly specific sequence enrichment, though realization of this potential will require further development. In particular, multiplexing of many probes into a single gel would enable simultaneous enrichment of multiple targets that would greatly enhance the usability of the tool. Although this would require careful probe design to minimize the interactions between probes and non-target DNA we feel this is not an insurmountable problem. We believe that the method of sequence and methylation enrichment presented here has the potential to be an enabling sample preparation technology for the analysis of rare nucleic acid targets for both research and clinical diagnostic applications.

## Methods

Experiments were carried out on a purpose built instrument similar to what was described in Pel et al [19]. The instrument was modified to include a two colour epifluorescence imaging system, and active gel temperature control. The gel thickness was reduced to 100 µm. Gels were composed of 4% polyacrylamide (49∶1 acrylamide∶bisacrylamide) with 10 µM concentration of acrydite modified oligonucleotides as capture probes (idtdna.com). Probe oligos were used as supplied by IDT without further purification. Target DNA for Figures 1, 2, 4, 6 and 7 were used as supplied by IDT without further purification. Target DNA for Figure 3 was obtained by PCR amplifying 250 bp and 1,000 bp regions of the pUC19 plasmid. Target DNA for Figure 5 was obtained by PCR amplifying a 460 bp region of the EZH2 gene from cDNA obtained from mutant and wild type cell lines [22].

The genomic DNA rejection experiments were done on a later iteration of the purpose built instrument, but functionally the same as the one described above. An E. coli culture was spiked into 1 ml of whole blood such that the total genome copy number ratio of E. coli to human was estimated to be roughly 1∶10. The mixture was lysed and purified using a protocol utilizing bead beating and magnetic beads, and phenol-chloroform extraction followed by high-salt purification. The purified DNA was sheared to below 1,500 bp with a sonicator, then diluted to 5 ng/ul in water. 1 ug (200 ul) of the sample was processed on the ssSCODA instrument at a time, and each run took approximately 4 hours. The 12.7 ul output from the ssSCODA process and the input sample were then quantified in a real-time quantitative PCR assay for the UidA gene of E. coli and for the GAPDH gene of human gDNA. A control run without spiking in the E. coli culture was also done.

Further details of the experimental apparatus and conditions are provided in File S1.

## Supporting Information

File S1 **Additional details regarding the experimental methods and calculations.**
(DOC)
