# Supplementary material for: Winnowing DNA for Rare Sequences: Highly Specific Sequence and Methylation Based Enrichment
Source: PLoS One. 2012 Feb 15;7(2):e31597. doi: 10.1371/journal.pone.0031597 (PMC3280224; doi:10.1371/journal.pone.0031597)
Supplement: File S1 — Additional details regarding the experimental methods and calculations. (DOC) [file pone.0031597.s001.doc]

# Materials and Methods

## Instrumentation

The instrument used for implementation of sequence specific SCODA (ssSCODA) is a modified version of instruments developed for electrophoretic SCODA [1-4]. The instrument was adapted to accommodate thin polyacrylamide gels, temperature control of the gel was added by placing the gel cassette onto a temperature controlled spreader plate, a two colour fluorescence imaging system was incorporated, and the voltage and current capabilities of the power supplies were increased. The instrument consists of a bipolar +/-500V 125W power supply (1/2C24-NP125, Ultravolt, Ronkonkoma, NY) with four independently controllable output voltage channels. Each output channel is based on a high voltage, high current operational amplifier (APEX PA94, Cirrus Logic Inc, Austin, TX) configured as an inverting amplifier with a gain of -40 V/V (32 dB), allowing the output channels to be driven from a +/-10V analog control signal. This power supply drives current through a custom designed gel cassette system fabricated with alternating layers of pressure sensitive adhesive and acrylic. Temperature control of the gel is achieved by placing the gel cassettes in contact with a temperature controlled aluminum spreader plate. The temperature of the spreader plate is measured with an RTD, and temperature is controlled with a high capacity thermoelectric chiller (HP-199-1.4-1.5, TE Tech, Traverse City, MI) mounted to a liquid cooled heat sink. The gel is imaged from above through a purpose built two colour epifluorescence imaging system capable of imaging fluorescein and Cy5 or similar dyes. The power supply, temperature control system and imaging system are all controlled through a computer running custom software written in LabView® (National Instruments, Austin, TX). A schematic of this system is shown in Figure S1.

Figure S1 Schematic of ssSCODA instrumentation.

In a later iteration of the instrument, a current measurement circuitry was added to the 4-channel high voltage amplifier and the software was modified so that the run duration can be specified by time or charge. In the charge duration mode, the software will turn off all amplifiers after a set amount of charge (product of current and time) has flown through one of the amplifiers in one direction, as specified by the user. Additionally, high-voltage relays were added in series between the amplifier and electrodes such that the electrodes could have a floating potential, and circuitry was added to increase the number of electrodes to six.

## Imaging System

A purpose built two colour epifluorescence imaging system was used to image the gels during injection and concentration. The system consists of two high intensity LED based excitation sources: a blue source centred at 450nm (LXML-PR01-350, Phillips Lumileds, San Jose California) for the excitation of fluorescien and similar dyes, and a red source centred at 632nm (LXML-PD01-0030, Phillips Lumileds, San Jose California) for the excitation of Cy5 and similar dyes. Light from each LED is collected and collimated by a condenser lens, the beams are cleaned up with excitation filters then combined with a short pass dichroic filter. Excitation light then passes through a secondary lens, is reflected off of a dual band dichroic beam splitter and through the object lens as shown in Figure S2 such that a uniform collimated beam emerges from the object lens. The emitted fluorescence is collected by the object lens then passes through the dual band dichroic beam splitter and is focused onto a CCD camera (Basler A102fc) by the image lens. Emission filters are mounted on a purpose build stepper motor driven filter wheel between the image lens and the CCD camera.

Figure S2 Two colour epifluorescence imaging system. Top: Schematic optical layout. Bottom: Image of actual system.

## Gel Cassette Construction

The polyacrylamide gels used for ssSCODA are cast within custom fabricated single use gel cassettes. The cassettes are comprised of three layers: a glass microscope coverslip is used as the bottom layer (Fisher Scientific P/N 12544F) which is in contact with the spreader plate, bonded to this is a 100μm thick layer of double sided pressure sensitive adhesive (MACtac P/N IP2100) which is laser cut to define the gel area. Bonded to the top surface of the PSA is a 1.5mm thick acrylic cover with access holes laser machined to enable electrical contact to the gel area. The cassettes are mated to a reusable buffer reservoir and sealed with a silicone gasket. Current is sourced through carbon electrodes placed in the buffer reservoirs. The details of the gel cassette assembly are shown below in Figure S3.


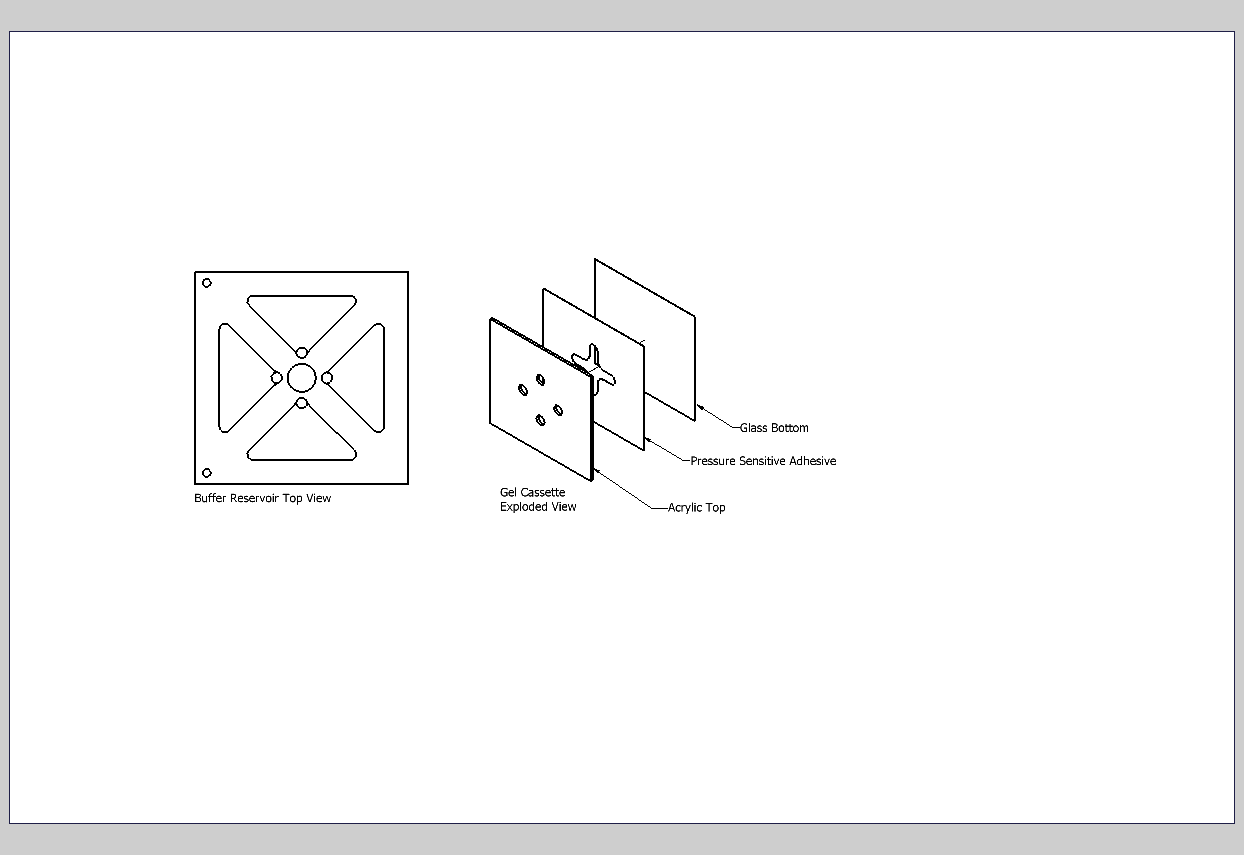

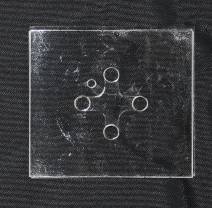

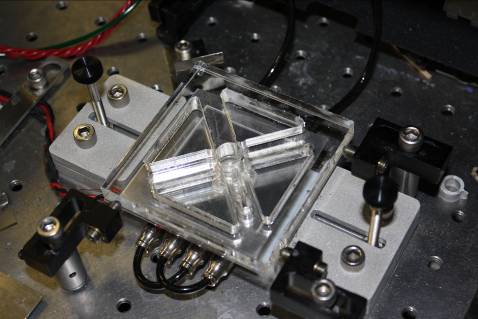


Figure S3 Gel cassette assembly. Top: schematic of the gel cassette and buffer reservoir assembly. Middle: top view of the buffer reservoirs and an exploded view of a gel cassette.. Bottom Left: Image of assembled gel cassette and buffer reservoirs on the spreader plate. Bottom Right: Gel cassette.

For DC mobility experiments the gel cassette was modified such that only two electrodes were in electrical contact with a gel area of constant cross section so that a constant electric field could be applied throughout the gel.

For genomic DNA rejection experiments, a cassette design which included an integrated sample chamber, an additional port for electrophoretic washing, and an extraction well was used, as shown in Figure S4.


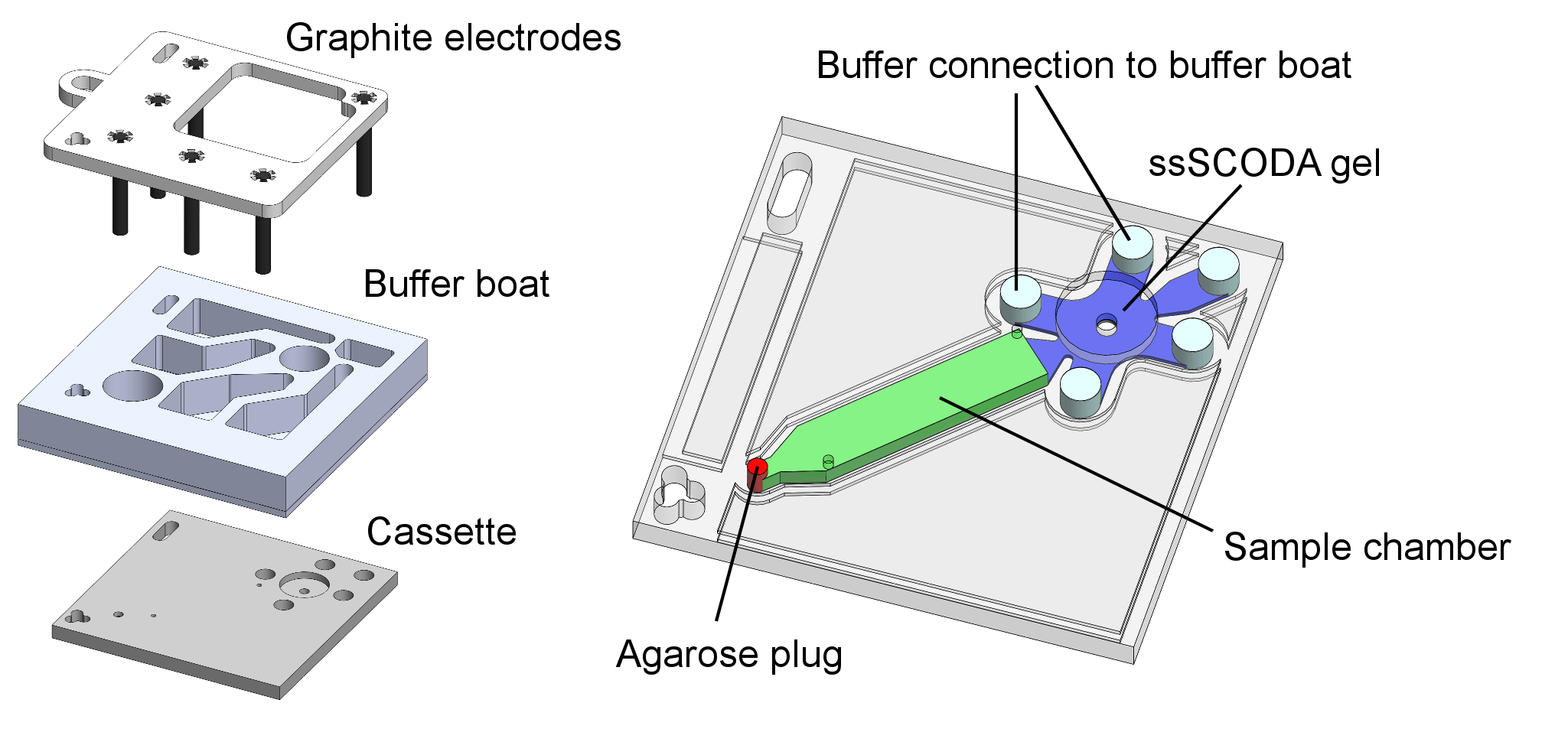


Figure S4 Schematic of the cassette assembly used in the genomic DNA rejection experiments. The sample chamber is integrated in the cassette.

## Gel Composition

All gels were composed of 4% acrylamide, 49:1 acrylamide/bis-acrylamide ratio (Sigma part number: A0924), in a solution of 89mM tris, 89mM boric acid, and 0.2M NaCl., pH 8.3 Acrydite modified probe oligos were included at a concentration of 20 μM for the genomic DNA rejection experiments, and 10 μM for all others. Prior to polymerization of the acrylamide gels 5mM sodium sulfite was added to scavenge oxygen and ensure complete polymerization against plastic surfaces. Polymerization was initiated with the addition of 20 μl of 10% w/v ammonium persulfate and 2 μl of TEMED per ml of gel.

## DNA Sequences Used

All probes and primers listed below, except for the MGB probe, were purchased from Integrated DNA Technologies, Inc. ([www.idtdna.com](http://www.idtdna.com/)), and were not purified in any way apart from the standard desalting offered by IDT. Probe oligos for ssSCODA concentration were ordered with the Acrydite modificiation. The MGB probe was purchased from Applied Biosystems ([www.appliedbiosystems.com](http://www.appliedbiosystems.com/)).

Probe sequence for single base mismatch and methylation enrichment experiments (Figures 1-3, 5 and 6):

5' ACT GGC CGT CGT TTT ACT 3'

100nt perfect match target sequence:

5' CGA TTA AGT TGA GTA ACG CCA CTA TTT TCA CAG TCA TAA CCA TGT AAA ACG ACG GCC AGT GAA TTA GCG ATG CAT ACC TTG GGA TCC TCT AGA ATG TAC C 3'

100nt single base mismatch target sequence

5' CGA TTA AGT TGA GTA ACG CCA CTA TTT TCA CAG TCA TAA CCA TGT AAA AC**T** ACG GCC AGT GAA TTA GCG ATG CAT ACC TTG GGA TCC TCT AGA ATG TAC C 3'

100nt perfect match methylated target sequence:

5' CGA TTA AGT TGA GTA ACG CCA CTA TTT TCA CAG TCA TAA CCA TGT AAA A**mC**G ACG GCC AGT GAA TTA GCG ATG CAT ACC TTG GGA TCC TCT AGA ATG TAC C 3'

Probe sequence for EZH2 enrichment (Figure 4):

5' AGT TTT CTG AGA TGA ATT CA 3'

Target DNA for the EZH2 experiments was PCR amplified from cDNA which was prepared from cell lines with either a wild type version of the EZH2 gene, or a version containing a Y641N mutant, as described in [5]. 460 bp regions of the cDNA were PCR amplified using the following primer sequences obtained from IDT:

Forward Primer:

5' TTA CTT GTG GAG CCG CTG ACC ATT 3'

Reverse Primer:

5' ATG CCG ACA TAC TTC AGG GCA TCA 3'

The forward primer was fluorescently tagged with Cy5 for the mutant sequence, and with 6-FAM for the wild type sequence.

Probe sequence for genomic DNA rejection experiments:5’ TTT ATA CCG AAA GGT TG 3’

Primer and probe sequences for the UidA real-time qPCR Taqman MGB assay:

Forward primer:

5'-GCCCAACCTTTCGGTATAAAGAC-3'

Reverse primer:

5'-GTTCGCCGATGCAGATATTCGT-3'

MGB Probe:

5’- 6FAM – TTCGCGCTGATACCAGAC 3’ MGB

Primer sequences for the GAPDH real-time qPCR SYBR assay:

Forward primer:

5’ GCA GGG CCT CAC TCC TTT 3’

Reverse primer:

5’ GGG CCA TCC ACA GTC TTC 3’

## SCODA Concentration:

## Sample Injection

To perform SCODA based concentration and enrichment samples are first electrokinetically injected into the gel by applying a constant electric field across a chamber adjacent to the concentration gel. Injection is most efficient when the sample salinity is considerably lower than the salinity of the gel. All DNA samples were suspended in a 250μl solution of 1mM tris, 1mM boric acid and 2mM NaCl, pH 8.3. An electric field of approximately 100V/cm was applied across the samples for approximately 5min at a temperature of 30°C.

## Concentration and Bias

Concentration is performed by applying a rotating electric field pattern as described in [4]. For all experiments the electric field was rotated stepwise through four discrete steps at a total period of 5 seconds (1.25 sec per step). To wash contaminating DNA from the gel a constant electric field is superimposed over the rotating field pattern. Table S1 shows the rotating voltage pattern applied to the four source electrodes, and Table S2 shows the applied voltages and run temperatures for each experiment.

|  | Electrode A | Electrode B | Electrode C | Electrode D |
| --- | --- | --- | --- | --- |
| Step 1 | $-V_{focus}$ | $\frac{V_{wash}}{2}$ | $V_{wash}$ | $\frac{V_{wash}}{2}$ |
| Step 2 | 0 | $-V_{focus}+\frac{V_{wash}}{2}$ | $V_{wash}$ | $\frac{V_{wash}}{2}$ |
| Step 3 | 0 | $\frac{V_{wash}}{2}$ | $-V_{focus}+V_{wash}$ | $\frac{V_{wash}}{2}$ |
| Step 4 | 0 | $\frac{V_{wash}}{2}$ | $V_{wash}$ | $-V_{focus}+\frac{V_{wash}}{2}$ |

Table S1 Focusing plus bias potentials applied to the gel. $V_{focus}$ is the focusing potential, and $V_{wash}$ is the wash potential

| Figure: | Focus Potential (V) | Wash Potential (V) | Temeprature (°C) |
| --- | --- | --- | --- |
| 2 | 90 | 7 | 62.0 |
| 3 | 120 | 10 and 20 | 62.0 |
| 4 | 60 | 7 | 62.0 |
| 5 | 132 | 10 | 59.0 |
| 6 (inset) and 7 | 84 | 3 | 69.4 |

Table S2 Concentration and wash conditions

## Image Analysis

All image analysis software was written in LabVIEW. The two color fluorescence images shown in Figures 2, 4 and 5 (inset) were taken one color at a time with the imaging system. Images were combined by extracting the green color plane from the image taken on the 6-FAM channel and the red color plane from the Cy5 channel and combining them into a new image.

## DC Mobility experiments

For the DC mobility experiments (Figures 1 and 5) a band of target DNA was injected into a gel containing bound probes and run at a constant electric field. Figure 1 was generated by applying a field 25V/cm, and Figure 5 was generated by applying a field of 10V/cm. Images were taken every 20 seconds and subsequently processed to determine the band position for each image. The position information was used to calculate velocity, and using the known electric field strength, mobility. The band position was calculated as the midpoint between the points in the image where the pixel intensity of the band was half of its maximum value.

## Rejection Ratio Experiments

To calculate rejection ratio the intensity of the final focus spot was determined on both the red and green channels and compared to a calibration run where a 1:1 ratio of 6-FAM and Cy5 labeled targets were concentrated to the centre of the gel. Specifically the measured rejection ratio, $S_{r}$, for a given input ratio of snMM:PC, $r$, was calculated as:

$$S_{r}=\frac{G_{r}}{G_{1}}\frac{R_{1}}{R_{r}}r$$

Where $G_{1}$ and $R_{1}$ are the fluorescence signals from the 1:1 calibration run on the red and green channels respectively, and $G_{r}$ and $R_{r}$ are the fluorescence signals on the green and red channels respectively after washing excess snMM target from the gel. The fluorescence signal was determined by integrating over a region of interest which contained the final focus spot and subtracting the background image intensity. The background intensity was taken from a region adjacent the final focus spot. For each experiment the same regions were used for both the red and green channels. These experiments were designed measure the rejection ratio while avoiding problems associated with non-linear relationships between fluorophore concentrations and fluorescence signals. By adjusting the amount of input snMM DNA until the final red to green signal ratio after washing was comparable to the 1:1 calibration run, errors associated with fluorophore self quenching, sensor noise, and sensor non-linearity can be minimized. Because of this, although all four experiments measured similar values for the rejection ratio, the most accurate measurement is the 10,000:1 experiment where the final signals from the green and red channels were most similar to the 1:1 calibration run. The total amount of DNA injected into the gel was chosen to ensure that the subsequent fluorescent signals, after concentration of PC and washing of snMM fragments, would not saturate the sensor.

| Run Description: | Cy5 Labeled Target | 6-FAM Labeled Target |
| --- | --- | --- |
| 1:1 Calibration | 10fmol PM | 10fmol PM |
| 100:1 | 1pmol sbMM | 10fmol PM |
| 1,000:1 | 10pmol sbMM | 10fmol PM |
| 10,000:1 | 100pmol sbMM | 10fmol PM |
| 100,000:1 | 1nmol sbMM | 10fmol PM |

Table S3 List of targets run for measuring the rejection ratio of ssSCODA with respect to single base differences. PM = perfect match. sbMM = single base mismatch.

# Genomic DNA Rejection Experiments

## Sample Preparation

For the genomic DNA rejection experiments, E. coli culture was spiked into blood. Whole blood (Center for Blood Research, UBC) was thawed to room temperature. *E. coli* DH10B (NEB) was sub-cultured in LB at 37 °C for 3 hrs from an overnight culture to obtain Logarithmic phase cells. 0.2 ul of the *E. coli* culture was added per ml of whole blood to obtain an approximate ratio of 1:100 genome copies (*E. coli*:Human). The bacterial-blood mixture was transferred to a 2 ml tube containing 1 g of Zr/Si beads (Invitrogen). 1 mg proteinase K (Roche) and 2.84% SDS (Invitrogen) was added per ml of blood. Cells were lysed in a beadbeater (MP Biomedical, P/N 116004500) for 5 cycles at 6 m/s for 1 min followed by 1 min resting, then incubated for 15 min at 60C. DNA was extracted from blood-bacterial mixture by adding equal volume phenol:chloroform:isoamyl alcohol (Sigma). The aqueous phase was re-extracted using equal volume of chloroform:isoamyl alcohol (Sigma). DNA in the aqueous phase was precipitated with 0.2 M sodium chloride and 0.7 volume of isopronanol and the pellet obtained was washed with 1 ml 70% alcohol. DNA was reconstituted in nuclease free water. Sodium chloride precipitation was repeated to eliminate residual impurities. Purified DNA was sheared to below 1.5 kb by sonication (Diagenode Bioruptor, P/N UCD-200TM-EX) at high setting for 20 minutes (30 seconds on, 30 seconds off) at 4°C.

## Pre-running gel.

After casting the gel in a cassette, the gel was pre-run so that any capture probes unincorporated into the gel matrix will be washed out of the gel. The running buffer (89 mM tris base, 89 mM boric acid, 0.2 M NaCl, pH 8.3) was loaded into the sample chamber and a DC voltage of 50 V at 30 °C and then 25 V at 75 °C was applied between electrodes E and F, for 1,000 mC each.

## Sample loading.

The sample chamber was rinsed with water three times, then the port closest to electrode E was plugged with a 2% w/v Seakem LE Agarose (Lonza P/N 50004) in running buffer. The purpose of this agarose plug was to prevent the sample mixing with the running buffer. 200 μl (1 μg) of the sample and 2.5 μl of the running buffer was pipetted into the sample chamber, and topped off with water to completely fill the sample chamber. The two pipet holes were sealed with small pieces of PCR sealing tape (Applied Biosystems, P/N 4311971).

## Sample Injection

A DC voltage of 150 V was applied between electrodes B and C, and electrode E, at a temperature of 30°C, for 1,500 mC. The temperature was raised to 75°C, and the same voltage pattern was applied at 50 V for 50 mC.

## Electrophoretic Washing

The sample chamber and extraction well were rinsed and filled with clean running buffer and sealed with PCR tape. A potential of 50 V was applied between electrodes E and F, at a set temperature of 30°C, for 750 mC. The extraction well was rinsed and filled with clean running buffer again, and the same field pattern was continued for an additional 750 mC. The sample chamber was then rinsed and filled with running buffer, and the extraction well was rinsed but sealed without refilling.

## Concentration and Extraction

A DC voltage of 25 V was applied in the same manner as the sample injection step, at 75°C for 750 mC. The concentration-with-bias field pattern as shown in Table S4 was applied for 20 minutes at 52 degC and at a voltage of 150 V. The extraction well was then filled and sealed with 6.3 ul of running buffer, and the concentration field pattern in Table S4 was applied for 20 minutes at 52 degC at a voltage of 150 V. Once complete, the buffer in the extraction well is extracted, and the well is rinsed with another 6 ul of running buffer to ensure full recovery. The collected buffer was weighed to determine exact volume.

|  |  | Electrode voltage | | | | | |
| --- | --- | --- | --- | --- | --- | --- | --- |
| Step | Duration (s) | A | B | C | D | E | F |
| 1 | 1 | 0 V | $V_{rail}$ | $V_{rail}$ | $V_{rail}$ | n/c | n/c |
| 2 | 1 | $V_{rail}$ | 0 V | $V_{rail}$ | $V_{rail}$ | n/c | n/c |
| 3 | 1 | $V_{rail}$ | $V_{rail}$ | 0 V | $V_{rail}$ | n/c | n/c |
| 4 | 1 | $V_{rail}$ | $V_{rail}$ | $V_{rail}$ | 0 V | n/c | n/c |
| 5 | 1 | n/c | n/c | n/c | n/c | $V_{rail}$ | 0 V |
| 6 | 1 | $V_{rail}$ | $V_{rail}$ | $V_{rail}$ | 0 V | n/c | n/c |
| 7 | 1 | $V_{rail}$ | $V_{rail}$ | 0 V | $V_{rail}$ | n/c | n/c |
| 8 | 1 | $V_{rail}$ | 0 V | $V_{rail}$ | $V_{rail}$ | n/c | n/c |
| 9 | 1 | 0 V | $V_{rail}$ | $V_{rail}$ | $V_{rail}$ | n/c | n/c |
| 10 | 1 | n/c | n/c | n/c | n/c | $V_{rail}$ | 0 V |

Table S4 Concentration-with-bias and concentration field patterns used for the genomic DNA rejection experiments. The concentration-with-bias field pattern is as shown while the concentration field pattern is without steps 5 and 10. Steps 5 and 10 are the wash steps. $V_{rail}$ refers to the rail voltage as described in text, and n/c means no connection.

## *qPCR Quantificatio*n

E. coli yield was quantitated using 625 nM primers (IDT) UidA-F: GCCCAACCTTTCGGTATAAAGAC and UidA-R: GTTCGCCGATGCAGATATTCGT and 100 nM MGB-probe (ABI) FAM-TTCGCGCTGATACCAGAC in a Taqman assay (Roche). Human DNA yield was quantitated using 200 nM primers (IDT) GAPDH-R: GCAGGGCCTCACTCCTTT and GAPDH-F: GGGCCATCCACAGTCTTC in a SYBR assay (ABI).

2 ul of each sample was assayed in a 50 ul reaction volume. DNA was denatured at 95C for 10 min and amplified for 40 cycles with 15s denaturation at 95C followed by and extension at 60C for 60s. Enrichment factor was calculated as the ratio between the *E.coli* yield and Human yield.

## qPCR Results

Figure S5 shows the results of qPCR quantification of ecoli and human DNA plotted against the standard curves. The values shown on the horizontal axis of Figure S5 are given as the copies per ml of 2ml aliquot added to the qPCR reaction. We estimate yield of the ssSCODA process by scaling the values quantified by the qPCR reaction by the initial volumes (200ul for the ssSCODA input, and about 12ul for the output) then taking the ratio of the total input DNA to the total output DNA, and multiplying by two since the input is single stranded and the output is double stranded. We found that the yield of *E. coli* DNA was approximately 20%. The enrichment ratio is given by the ratio of the e.coli yield to the human yield. The quantitation of the human DNA output fell well outside of the standard curve, so direct quantitation of the enrichment ratio was not possible. Using the lowest point on the standard curve as an upper limit for the amount of human DNA present in the output, we can say that the enrichment factor was greater than 152 fold. Extrapolating the standard curve gives an enrichment ratio on the order of 10^3^, however this value has considerable uncertainty.

Figure S5 Quantification of human and E. coli DNA before and after ssSCODA purification. The horizontal axis represents the number of DNA copies per μl of sample added to the PCR reaction. 2 μl were added to each PCR reaction from a total sample volume of 200 μl for the input samples and 12.7 μl for the output samples. Top: The UidA gene was used for E. coli quantification. Bottom: GAPDH was used for human quantification. Note that the GAPDH no-template control (NTC) did not amplify. These results suggest that we recovered 20% of the UidA strands that were complementary to the gel probes, with an enrichment factor greater than 152 fold. Extrapolating the GAPDH standard curve suggests an enrichment factor on the order of 10^3^, however this is an estimate with considerable uncertainty.

# DNA Mobility in an Affinity Matrix

In this section we will discuss the electrophoretic mobility of a target DNA molecule moving through an affinity matrix containing immobilized probe DNA molecules which are complementary to the target, and show how one can perturb the mobility of a DNA molecule moving through such a matrix.

The interactions between the target and immobilized probes can be described by first order reaction kinetics:

Here $[T]$ is the target, $[P]$the immobilized probe, $[T\cdots P]$ the probe-target duplex, $k_{f}$ is the forward (hybridization) reaction rate, and $k_{r}$ the reverse (dissociation) reaction rate. Since the mobility of the target is zero while it is bound to the matrix, the effective mobility of the target will be reduced by the relative amount of target that is immobilized on the matrix:

$$\mu_{\text{effective}}=\mu_{0}\frac{[T]}{[T]+[T\cdots P]}$$

Where $\mu_{0}$ is the mobility of the unbound target. If we assume that the binding kinetics are fast compared to the period of our perturbing field then we can rewrite equation in terms of reaction rates:

$$k_{f}[T][P]=k_{r}[T\cdots\text{P]}$$

$$[T]=\frac{k_{r}[T\cdots P]}{k_{f}[P]}$$

Inserting into equation and simplifying we get:

$$\mu_{effective}=\mu_{0}\frac{1}{1+\frac{k_{f}}{k_{r}}[P]}$$

From this result it can be seen that the mobility can be altered by modifying either the forward, or reverse reaction rates. The simplest approach to modifying the mobility of DNA targets moving through an affinity matrix is through control of the matrix temperature.

To show how this is possible it is helpful to make some simplifying assumptions. First we assume that we have a large number of probes relative to target molecules, so long as this is true then even if a large fraction of the target molecules become bound to the probes the concentration of free probes, $[P]$, will not change much and we can assume that $[P]$ is constant. Also, we assume that the forward reaction rate $k_{f}$ does not depend on temperature. This not strictly true, as the forward reaction rate does depend on temperature [6,7], and secondary structure in the probe or target sequence can result in a temperature dependant forward reaction rate [8]. However we will be operating in a regime near the duplex melting temperature where the reverse reaction rate has an exponential dependence on temperature and the forward reaction rate has a much weaker temperature dependence, varying by about 30% over a range of 30°C around the melting temperature [9], and additionally assume that the target sequence is free of any significant secondary structure. To determine the temperature dependence of the reverse reaction rate we assume an Arrhenius model for unbinding kinetics which is justified by recent work in nanopore force spectroscopy [10,11].

$$k_{r}=Ae^{\frac{\Delta G}{k_{b}T}}$$

Here $A$ is an empirically derived constant, $\Delta G$ is the probe-target binding energy, $k_{b}$ is the Boltzmann constant, and $T$ the temperature. Inserting this into , rewriting the free energy $\Delta G$ as $\Delta H-T\Delta S$, and collecting constant terms we can rewrite the mobility as:

$$\mu_{\text{effective}}=\mu_{0}\frac{1}{1+\alpha e^{\frac{-\Delta H+T\Delta S}{k_{b}T}}}$$

1. Broemeling DJ, Pel J, Gunn DC, Mai L, Thompson JD, et al. (2008) An Instrument for Automated Purification of Nucleic Acids from Contaminated Forensic Samples. JALA 13: 40-48.

2. Marziali A, Pel J, Bizzotto D, Whitehead LA (2005) Novel electrophoresis mechanism based on synchronous alternating drag perturbation. Electrophoresis 26: 82-90.

3. Pel J (2009) A novel electrophoretic mechanism and separation parameter for selective nucleic acid concentration based on synchronous coefficient of drag alteration (SCODA). Vancouver: University of British Columbia.

4. Pel J, Broemeling D, Mai L, Poon H-L, Tropini G, et al. (2009) Nonlinear electrophoretic response yields a unique parameter for separation of biomolecules. Proceedings of the National Academy of Sciences: -.

5. Morin RD, Johnson NA, Severson TM, Mungall AJ, An JH, et al. (2010) Somatic mutations altering EZH2 (Tyr641) in follicular and diffuse large B-cell lymphomas of germinal-center origin. Nature Genetics 42: 181-U124.

6. Wetmur JG, Davidson N (1968) Kinetics of Renaturation of DNA. Journal of Molecular Biology 31: 349-&.

7. Meinkoth J, Wahl G (1984) Hybridization of Nucleic-Acids Immobilized on Solid Supports. Analytical Biochemistry 138: 267-284.

8. Tsourkas A, Behlke MA, Rose SD, Bao G (2003) Hybridization kinetics and thermodynamics of molecular beacons. Nucleic Acids Research 31: 1319-1330.

9. Craig ME, Crothers DM, Doty P (1971) Relaxation Kinetics of Dimer Formation by Self Complementary Oligonucleotides. Journal of Molecular Biology 62: 383-&.

10. Nakane J, Wiggin M, Marziali A (2004) A nanosensor for transmembrane capture and identification of single nucleic acid molecules. Biophysical Journal 87: 615-621.

11. Nakane JJ (2006) Detection and analysis of nucleic acids using nanometer-scale pores. Vancouver: University of British Columbia.
